# Analysis of merged transcriptomic and genomic datasets to identify genes and pathways underlying residual feed intake in growing pigs

**DOI:** 10.1038/s41598-022-26496-1

**Published:** 2022-12-19

**Authors:** Emil Ibragimov, Anni Øyan Pedersen, Liang Xiao, Susanna Cirera, Merete Fredholm, Peter Karlskov-Mortensen

**Affiliations:** 1 Department of Veterinary and Animal Sciences, Faculty of Health and Medical Sciences, University of Copenhagen, 1870 Frederiksberg C, Denmark; 2 Metagenomic Institute, BGI Research, GeneBank, Jinsha Road, DaPeng District, Shenzhen, 518083 Guangdong China

**Keywords:** Data processing, Gene regulatory networks, Animal breeding, Functional genomics, Gene expression, Genetic association study, Genome

## Abstract

Improvement of feed efficiency (FE) in pigs is an important milestone in order to reduce the economic and environmental impact of pig production. The goal of finding biomarkers for FE has persisted for decades. However, due to the complexity of the FE trait, these goals have still not been met. Here, we search for quantitative trait loci (QTL), candidate genes, and biological pathways associated with FE using both genotype and RNA-seq data. We obtained genotype and colon epithelium RNA-seq data for 375 and 96 pigs, respectively. In total, a genome-wide association study (GWAS) and differential expression (DE) analysis led to detection of three QTL on SSC9 and 17 DE-genes associated with FE. Possible intersection points between genes located in QTL and DE-genes were found on levels of transcription factor-target interaction. Moreover, cis-eQTL analysis revealed associations between genotype and expression levels of three DE-genes and three genes located in the GWAS QTLs, which may establish the connection between genotype and phenotype through DE. Finally, single nucleotide polymorphism calling using RNA-seq data for genes located in GWAS QTLs revealed 53 polymorphisms of which eleven were missense variants.

## Introduction

As the human population grows, the requirement for effective food production industries intensifies. At the same time, environmental challenges require food production to be sustainable. Extensive agriculture expansion has already led to the usage of one half of habitable land, significant changes in the landscape^1^ and numerous other challenges, such as greenhouse gas emission, terrestrial acidification, and freshwater eutrophication^2^. Life cycle assessment studies have shown that feeding accounts for 28–82% of the total impact on climate change from animal production^3^ and that breeding for feed efficiency (FE) traits can reduce this impact^4^. This has raised new arguments for improvements in FE in pigs.

FE in pigs is a complex trait with a large number of biological and environmental components. Environmental factors such as feed composition are constantly studied, leading to new understanding and advances in the optimization of feed composition^5–7^. Furthermore, advancements in molecular biology techniques have led to several studies directed at identifying the biological components involved in FE in pigs. Most of these studies^8–12^ have been aimed at finding biological markers for FE in pigs using different techniques such as genetic mapping, RNA-sequencing, metabolomics, and metagenomics.

Among these studies, genome-wide association studies (GWAS) are especially interesting, since GWAS results can aid in the understanding of genetic mechanisms for high FE. However, a main drawback is that the heritability of FE is quite low, thus a cohort for a GWAS on this trait must be big to gain enough statistical power for detection of quantitative trait loci (QTL)^13^. Another issue of GWAS is that in most cases it is not possible to infer the location of causative mutations precisely, thus a list of candidate genes potentially associated with a trait prevails after GWAS. A potential way to overcome this obstacle is to include another omics analysis since consistent results of GWAS and other omics analysis can be regarded as mutual verification.

Omics techniques have found widespread use in porcine FE research, in combination with GWAS or on their own. A number of associations with different types of biological markers have been found in these studies, which overall provide a better understanding of the FE trait complexity. For instance, metagenomics research has shown an association of dozens of bacterial taxonomic units in gut microbiota with FE^8,11^, and metabolomics studies in combination with transcriptomic^9^ and GWA studies^10^ have revealed significant gene-metabolite pairs. Transcriptomic studies have found FE associated differentially expressed genes (DE-genes) in various tissue, including blood^14^, liver^12^, brain^15^ and intestine^16^.

The colon is a part of the gastrointestinal tract that absorbs water, electrolytes and vitamins from the digesta as it arrives from the small intestine. Furthermore, the colon provides space for a dense and diverse community of mainly anaerobe bacteria with the ability to ferment complex carbohydrates, which otherwise cannot be digested by the host. The bacterial fermentation results in synthesis of short chain fatty acids, which are a rich source of energy for the host^17,18^. The healthy symbiotic relationship between host and microbiota is tightly regulated by multiple factors and hence, gene expression studies focusing on colon epithelium cells may disclose new aspects of the host-microbiota symbiosis.

Residual feed intake (RFI) can be used to describe FE in pigs. It is calculated as a difference between observed feed intake and expected feed intake as predicted by multiple regression of feed intake on production traits such as body weight gain, and tissue composition to account for production requirements, and average metabolic body weight to account for maintenance requirements^19^.

In the present study, we combine transcriptome analysis and GWAS to identify candidate genes and pathways linked with FE measured as RFI. Both types of analysis reveal biological markers for FE.

## Results

### Genome-wide association studies

GWAS was performed using 370 pigs after filtering outlier pigs as described in the methods section. The test model included RFI values (MJ NE/d) as response variable and sex as a fixed effect. Mean and standard deviation for RFI were 0 and 1.111, respectively, and RFI was strictly normally distributed according to the Shapiro–Wilk test. The heritability for RFI was 0.17.

**Figure 1:**
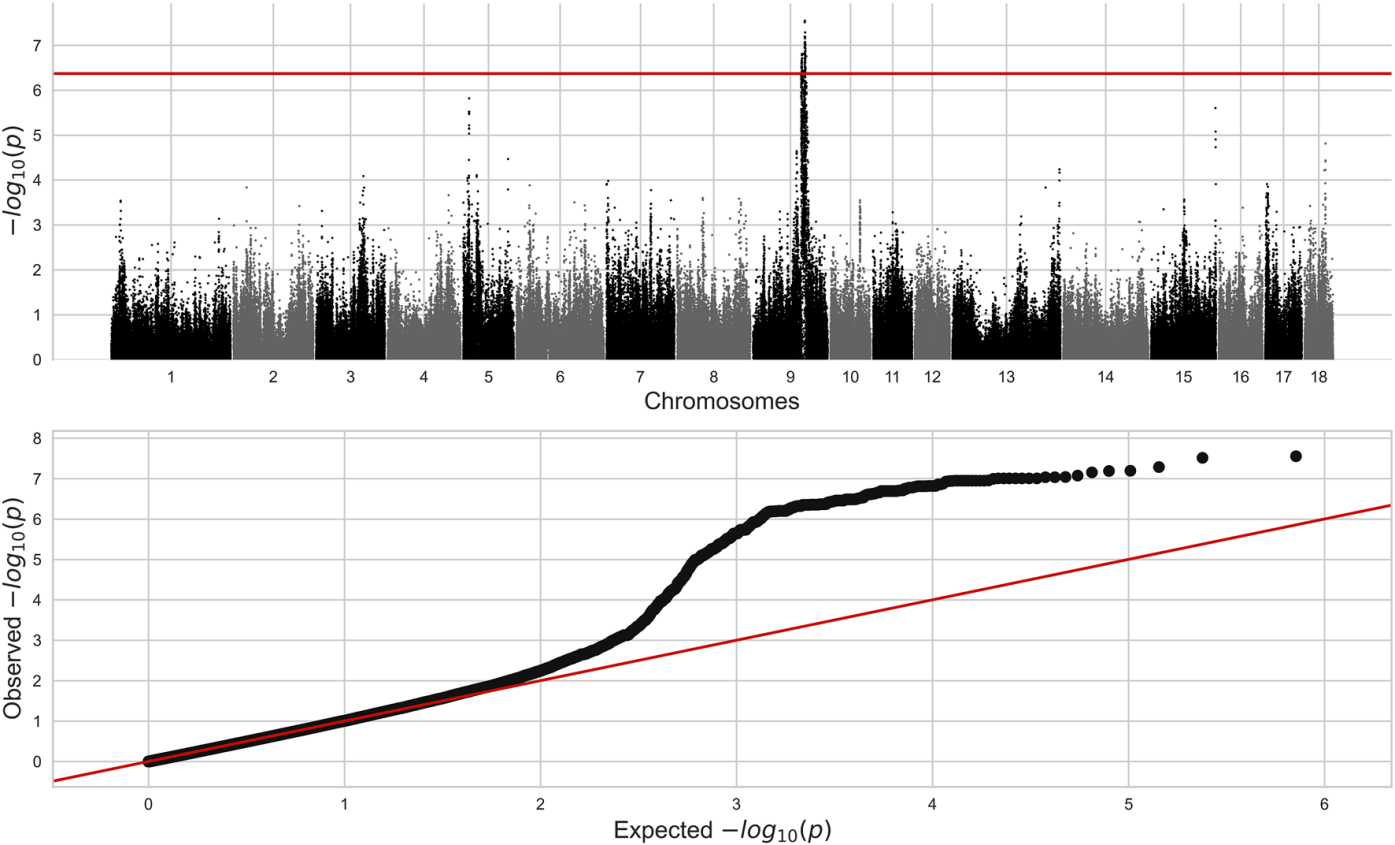
Manhattan (top) and Q-Q (bottom) plots of GWAS results.

Results of the GWAS are shown in Fig. 1. Genome-wide significance threshold was 4.28e-7 after Bonferroni correction. All genome-wide significantly associated SNPs were located in a region on SSC9 from 83.7 to 95.9 Mb (Table 1). A significant deviation in observed* p-*values as compared to expected* p-*values is evident in the qq-plot. This reflects a large number of markers in strong LD with the genome-wide significant SNPs on SSC9.

**Table 1:** Description of QTL blocks as defined by linkage disequilibrium.

	Location	Lead SNP	*p*-value	Number of genes	Number of protein coding genes	Genes flanking the lead SNP
QTL-1	SSC9: 83,701,293–86,854,470	9:85907846	1.54e-7	24	15	BZW2
QTL-2	SSC9: 88,546,817–91,460,506	9:90703434	2.80e-8	19	13	DNAH11
QTL-3	SSC9: 95,697,312–95,866,333	9:95864157	1.76e-7	0	0	*ENSSSCG00000042154 *ENSSSCG00000042154

*Flanking genes for lead SNP in QTL-3 are located outside the QTL.

All except three SNPs associated with RFI were located in a 10 Mb region on SSC9, which subsequently was studied for LD patterns (Suppl. Figure 1). The LD analysis revealed the majority of SNPs to be located in two LD blocks, SSC9: 83,701,293–86,854,470 (QTL-1) and SSC9: 88,546,817–91,460,506 (QTL-2), whereas one SNP was located outside the two LD blocks in an LD block located at SSC9: 95,697,312–95,866,333(QTL-3). Hence, the LD analysis resulted in identification of three QTLs, two relatively wide (QTL-1 and QTL-2) and one narrow (QTL-3) (Table 1, Suppl. Figure 1).

QTL-1 contained fifteen protein-coding genes and eight non-coding genes, and QTL-2 contained thirteen protein-coding and six non-coding genes (Suppl. Table 1). Inside QTL-3 no genes were found but the nearest flanking genes are given in Table 1.

**Figure 2:**
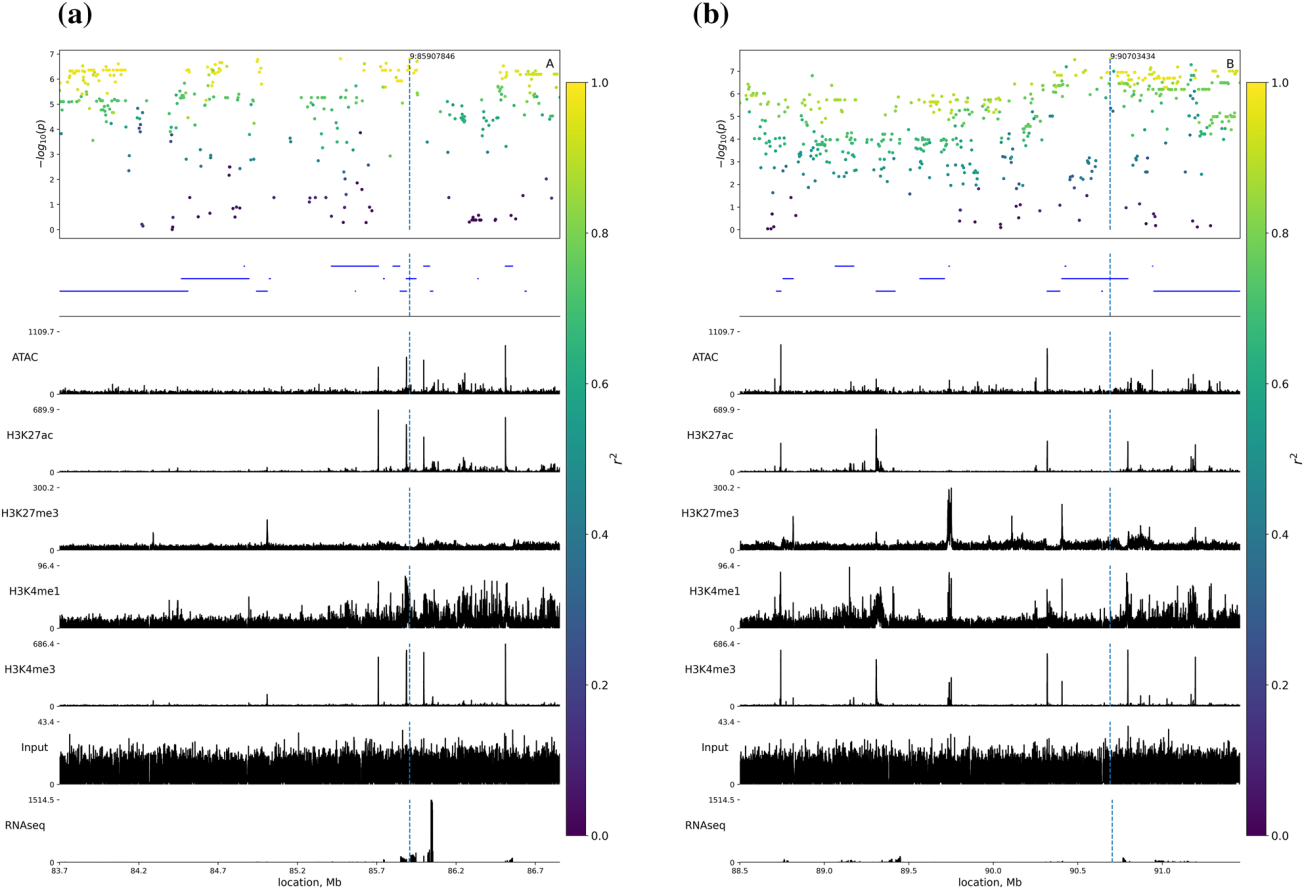
Plots of QTL-1 (**a**) and QTL-2 (**b**) GWAS results, gene location, and epigenetic markers enrichment. X-axes indicate genome positions, Y-axis in the top panel indicates − log (*p* values). Linkage disequilibrium between the lead SNP and other SNPs is highlighted by color. Blue lines represent the location of genes, the remaining plots illustrate epigenetic marker enrichment scores. Score values on Y-axes correspond to aligned read counts in the ChIP-seq analysis reported by Pan et al. 2021^20^.

The lead SNP from the QTL-1 block was located in an intron of *BZW2* in a region enriched for ATAC-, H3K27ac-, and H3K4me1-signals in colon tissue^20^ indicating a regulatory role of this region (Fig. 2a).

The lead SNP from QTL-2 was located in an intron of the *DNAH11* gene. The location of this lead SNP was not enriched for epigenetic markers in colon tissue (Fig. 2b).

### Analysis of differential expression

For analysis of differential gene expression (DE-analysis) RNA sequencing data of colon epithelium was obtained from 96 preselected pigs with high or low RFI as described in the methods section. Four pigs were excluded from the analysis because they were found to be outliers with regard to their RFI based on statistics described in the methods section. Furthermore, one pig was found to be an outlier in respect to gene expression and was excluded from further analysis. Hence, after filtering, 47 (24 females and 23 males) low RFI pigs and 44 (22 females and 22 males) high RFI pigs were left for DE-analysis. For these pigs, mean values of RFI for the low and high RFI groups were − 1.56 and 1.56, respectively, standard deviation within groups were 0.57 and 0.88, respectively. The distribution of* p-*values for the DE model was anti-conservative (Suppl. Figure 2).

**Figure 3:**
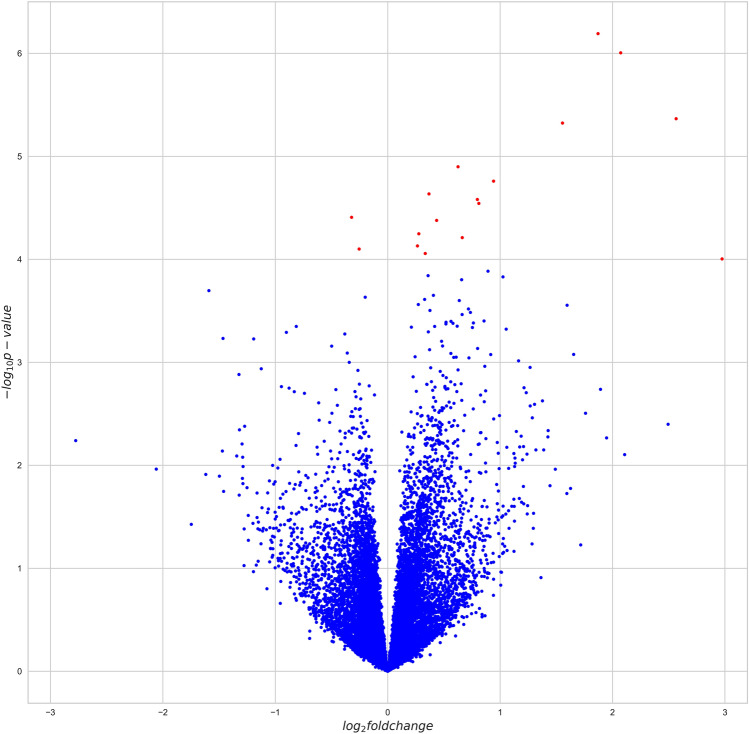
Differentially expressed genes between the high RFI and the low RFI. Red dots represent significantly up- or downregulated genes.

A False Discovery Rate of 0.1 was used as a threshold for significance in the DE-analysis (Fig. 3). This resulted in identification of 17 DE-genes with 15 genes upregulated in the high RFI group and two genes downregulated in the high RFI group (Suppl. Table 2). Immune system associated terms were found in the rank-based enrichment test for* p-*values of all genes tested in the DE-analysis. These functions include regulation of T-cell activation, regulation of leukocyte cell–cell adhesion, and antigen processing and presentation (Suppl. Table 3). The same type of enrichment test but for log fold change values of genes showed that immune system associated terms were characteristic for upregulated genes in the high RFI group (Suppl. Table 4). Downregulated genes were characterized by a diverse group of terms, including terms related to mitosis, protein synthesis, etc.

### eQTL analysis

For the eQTL analysis, 94 pigs with both RNA-seq and genotype data available were used. Only cis-eQTL mapping was performed and SNPs located no more than 1 Mb from a gene were considered. Significance threshold was set based on a three step correction procedure as described by Huand et al*.*^21^. Genes and SNPs passing the significance threshold are called eGenes and eQTLs, respectively in the following.

In total, 2016 eGenes were identified and their expression was associated with 5263 different cis-eQTLs. The 2016 eGenes represent: 1701 protein-coding genes, 269 lncRNA genes, 35 pseudogenes, 3 snRNA genes, 3 processed pseudogenes, 2 IG V genes, 1 miRNA gene, 1 snoRNA gene, and 1 TR V gene.

eQTL were found for two out of the 17 DE-genes, *ENSSSCG00000025271*, and *ENSSSCG00000034614*. None of these eGenes had a paired eSNP with a* p-*value in GWAS lower than 0.05.

Three eGenes, *AGMO*, *SOSTDC1*, and *CRPPA*, were found in QTL-1 (Table 2). Only *AGMO* had a relatively low* p-*value in the DE-analysis.

**Table 2:** eGenes among Differentially Expressed RFI QTL genes.

eGene	eSNPs	baseMean	log2FoldChange	P_DE_	FDR_DE_
AGMO	9:84047949	3.41	−1.27211	0.00418	0.41600
CRPPA	9:85565144	32.77	0.26116	0.15780	0.81436
SOSTDC1	9:84672568	8.15	−0.67361	0.19125	0.84661

### SNP calling using RNA-seq data

SNP calling revealed 53 polymorphisms in genes located in QTL-1 and QTL-2. Using the Ensembl Variant Effect Predictor tool^22^ it was found that eleven polymorphisms were missense variants (Suppl. Table 5). Among those, two variants were deleterious based on SIFT score, both of them were located in a conserved domain of the *DNAH11 *gene. Moreover, four missense mutations were located inside the *AHR* gene.

### Transcription factor regulation

**Figure 4:**
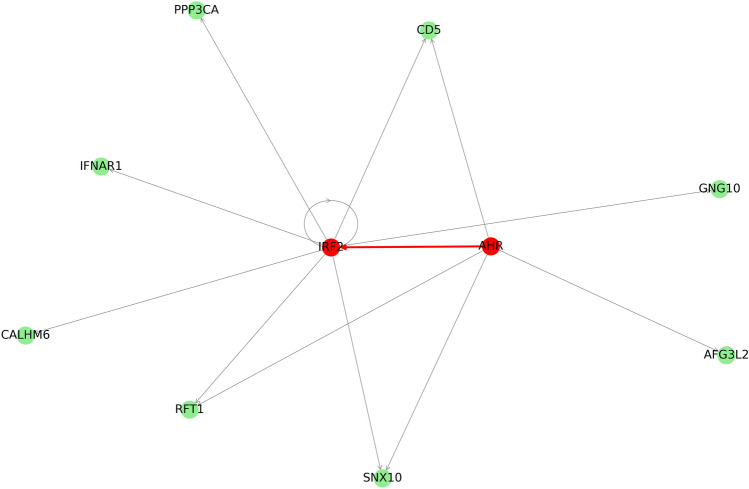
Graph of transcription transcription factor regulation pathways among DE-genes (green) and of RFI QTL gene (red). Interactions between transcription factors highlighted by red color.

A survey of the GeneHancer database revealed that the AHR transcription factor encoded by a gene located in QTL-1 has five targets among proteins encoded by DE-genes (Fig. 4). Among target DE-genes for AHR, one transcription factor was found. This transcription factor, IRF2*, *in turn, regulates the expression of four additional DE-genes.

## Discussion

The combination of GWAS and RNA-seq data analysis provided an opportunity for making a multi-faceted analysis of biological markers for RFI. Using the two methods in tandem, we obtained mutual support for results from multiple analyses.

The priority in our study was the identification of QTLs for RFI and the biological pathways on which they have an impact, thus RNA-seq data was mostly used for verification and interpretation of GWAS results. We have achieved this in three different ways: identification of transcription factors in QTLs with targets in DE-genes, finding cis-eQTLs in QTL regions for RFI, and SNP calling based on RNA-seq data.

The present GWAS analysis revealed three QTL regions on SSC9, which overlap with a number of QTL found in previous studies according to the Animal QTL Database^23^. In total, QTLs from 26 previous studies are overlapping with the QTLs identified in the present study. Two of these overlapping QTLs are associated with production traits such as body weight at day 21 (QTL-1,2,3)^24^, lipid (QTL-2) and protein (QTL-1,2,3) accretion rate, and average daily gain (QTL-1,2,3)^25^. However, since FE is a genetically highly complex trait, the QTLs identified in the present study are at best small pieces in the genetic puzzle underlying FE. A more complete picture of the genetic mechanisms underlying this trait must include the many other QTLs found in previous studies on this subject [e.g.^13,26,27^].

In our GWAS analysis the lead SNP for QTL-1 was located in a region enriched for ATAC-, H3K27ac-, and H3K4me1-signals in colon tissue. The lead SNP for QTL-2 was located in an intron region of the *DNAH11* gene. A potential functional importance of this gene is supported by SNP calling based on RNA-seq data which revealed two deleterious variants in conserved domains of *DNAH11*. SNPs located in QTLs were associated with the expression of three genes, however, only one gene, *AGMO*, shows a* p-*value in the DE-analysis lower than 0.05.

Analysis of DE in the RNA-seq data revealed 17 DE-genes for RFI. Fifteen DE-genes were upregulated in the high RFI group of pigs. Rank-based enrichment test for log fold change values showed that expression upregulation in the high RFI group was enriched for immune system associated annotations in agreement with previous studies^14,28^. The identified immune system response in colon epithelium might indicate a link between gut immune function, the gut microbiome and FE. The causation of this link can be explained in two different ways. On one hand, the microbiome might modulate immune system response. For example, it has been shown that short-chain fatty acids (SCFAs) are involved in immune response inhibition^29,30^. Energy rich SCFAs are also product of fiber fermentation by the gut microbiome and therefore increase of their production might lead to increased FE. On the other hand, a modified immune response might be determined genetically and subsequently modulate the microbiome. The potential role of genetics in this link is supported by interactions found between GWAS and DE results using transcription factor–target networks. In this way, a transcription factor encoding gene located in QTL-1, *AHR*, may explain the differential expression of nine out of 17 DE-genes. The *AHR* gene has to our knowledge not been linked to FE before, but it has been considered a candidate gene for reproductive traits in previous studies^31–33^. SNP calling based on RNA-seq data revealed four missense mutations in the protein-coding part of the *AHR* gene. Thus, we conclude that *AHR* is a promising candidate gene for a RFI genetic determinant.

Moreover, one gene located in QTL-1, *AGMO*, showed an expression level significantly associated with the genotype and a relatively low* p-*value in DE-analysis for RFI. This gene encodes alkylglycerol monooxygenase which appears to play a role in immunity, energy homeostasis, and development^34^. In our study *AGMO* was down-regulated in the high RFI group of pigs.

In conclusion, we have identified a number of genes of potential importance for RFI. Of particular interest is the gene encoding the AHR transcription factor, which is located in QTL-1 and targeting some of the identified DE-genes. Another gene of interest is *DNAH11*, in which the QTL-2 lead SNP was located in one of the introns. Missense mutations were found in both *AHR* and *DNAH11* genes. Further studies in independent cohorts should be performed to confirm the relevance of the candidate genes for feed efficiency in pigs. Furthermore, the hypotheses concerning interactions between immune system response in colon and microbiome could be studied by integration of metagenomics data. Such data will soon be available for the pigs in the present study and an analysis of these data will be presented in a separate paper.

## Materials and methods

### Animals

In the present study, 409 cross-bred animals were used. Study animals were produced by insemination of (Landrace x Yorkshire) crossbred sows by mixed semen from Duroc boars. DanBred (Herlev, Denmark) provided all animals. Conditions from the Danish “Animal Maintenance Act” (Act 432 dated 09/06/2004) and the “Order regarding animal experimentation” (BEK nr 12 af 07/01/2016) were met and approved by the Danish Veterinary and Food Administration. Individual IDs were assigned to each pig at weaning and each pig was equipped with an electronic ear-tag. An automatic feeding station was used to record the time, duration, and feed consumption at every visit of the pig using ear-tag screening. The trial period started when pigs reached the approximate body weight of 30 kg and lasted for 33 days. The same diet was given to all pigs. None of the pigs were treated during the trial period. The diet was composed of wheat (49%) barley (25%) and soybean meal (17%). The crude protein (CP) content was 16% and the content of standardized ileal digestible protein was 132 g per kg feed. The energy content was 9.6 MJ NE per kg feed. Individual feed intake was measured in MJ NE. All pigs were slaughtered after overnight fasting at an age of approximately 6 months and a bodyweight of approximately 100 kg in a commercial abattoir. Blood from each pig was sampled in Thermo Fischer BD K2E (EDTA) tubes immediately after exsanguination.

### DNA isolation, genotyping and imputation to whole genome sequence variants

High quality DNA was isolated from EDTA stabilized blood using a classic salting out procedure^35^. SNP genotyping was performed by Edinburgh Genomics, Ashworth Laboratories (Edinburgh) using the 700 K Affymetrix Axiom PigHD chip. Sequences of the probes for Affymetrix Axiom PigHD SNPs were mapped to the new assembly using BWA^36^ to retrieve marker positions in the newest version of the pig genome assembly (Sscrofa11.181). Markers that were assigned to multiple positions across the genome were excluded from the analyses. Only autosomal markers were retained. The haplotype phasing for the HD marker set was performed using Eagle^37^ with default parameters. Finally, Minimac3^38^ was used to impute missing values of the HD marker set. Imputation of missing data was aided by exploitation of DNA sequence information from 217 purebred pigs of the three involved breeds.

### Phenotype calculation

Residual feed intake (RFI) was defined as the difference between an observed average daily feed intake (ADFI) and expected ADFI. Expected ADFI was estimated using multiple linear regression of ADFI on average daily gain and metabolic body weight (MBW). MBW was calculated under an assumption of a linear growth rate over the experiment period using formula from^39^:

$$MBW=\frac{({W}_{1}^{1.75}-{W}_{0}^{1.75})}{1.75\times ({W}_{1}-{W}_{0})}$$

where W_1_ and W_0_ are weights at start and end of the test, respectively.

The linear model was built using the LinearRegression function in the Sklearn package V.1.1.2^40^. At first, data for 409 pigs were used, however nine pigs were excluded from calculation of expected ADFI and further analysis as outliers. Outlier pigs were identified using Cook’s distance values in the abovementioned multiple linear regression model. Cook’s distance estimates the impact of separate data points in a least squares regression and identify extreme observations for elimination^41^. Cook’s distance threshold was set on the level of the 98th percentile (Suppl. Figure 3). Outliers were excluded prior to building the final linear model used for RFI calculation.

The 96 pigs selected for RNA sequencing (see below) contained four outliers, thus 92 pigs were left after filtering.

### Genome-wide association study

375 pigs were genotyped, but 5 pigs were excluded from GWAS as phenotype outliers as described above. Quality control for the genotypes was conducted based on the criteria of Hardy–Weinberg equilibrium (HWE > 10^–8^) and minor allele frequency (MAF > 0.05) by PLINK software^42^. After quality control, 357,735 SNPs remained for further analysis. We estimated the genomic relationship matrix (GRM) using GCTA^43^ with the filtered autosomal marker set. GWAS analyses were performed using the “leave-one-chromosome-out” procedure in GCTA. Number of independent tests (N_indep_) was estimated using SimpleM^44^ and a Bonferroni corrected genome wide significance level was defined as 0.05/N_indep_. Genome-wide significance threshold was 4.28e-7 after Bonferroni correction.

GWAS was performed for each chromosome using RFI as a response phenotype and including sex as a fixed effect. Genome-wide significantly associated SNPs were further used for QTL region identification.

QTL regions were identified based on linkage disequilibrium (LD) between lead SNPs in a region and surrounding SNPs. Pairwise LD (r^2^) was calculated using the –r2 function in PLINK^42^. Borders of QTLs were defined based on position of the most distant SNP from the lead SNP with an r^2^ score above 0.85.

Genomic heritability was estimated for RFI using sex as a fixed effect. For estimation, the GCTA –reml function and the GRM established above were used.

Identified QTL regions were screened for epigenetic marker tracks in colon tissue based on published pig genome functional annotations^20^. Epigenetic marker tracks were obtained from the UCSC database^45^ and visualized based on scores. Scores were derived from ChIP-seq data and represent processed counts of reads aligned to the given location in the genome.

### RNA extraction and RNA sequencing

At a commercial slaughterhouse, the gastrointestinal organs were removed from the pigs within 20 min after bleeding. Approximately 5 cm of the middle part of the colon was cut out and rinsed in 0.9% saline. The mucosal lining was scraped off with a scalpel, transferred to cryo-tubes, and snap-frozen in liquid nitrogen. Samples were collected from 325 pigs. Among those pigs, 48 animals with high FE and 48 animals with low FE were selected for RNA sequencing. An equal number of females and males were selected for both high and low FE. The animals were selected based on feed conversion ratio (FCR) adjusted to the weight of pigs at the start of the trial. Mean values of adjusted FCR in high and low FCR groups were 2.291 and − 1.799 MJ NE/kg, respectively. The mean and standard deviation value for adjusted FCR in the entire population were 0 and 1.593, respectively. Thus, a ratio of sample difference between groups to standard deviation in the entire population was 2.567.

For RNA isolation, 50 mg of tissue per sample were homogenized in a GentleMACS™ Octo Dissociator machine (Miltenyi Biotec) using the RNeasy® Mini Kit (Qiagen) with DNase digestion, following manufacturer’s instructions. The concentration and purity of the RNA samples were measured on a Nanodrop ND-1000 spectrophotometer (NanoDrop Technologies, Wilmington, USA). RNA integrity was assessed on a BIO-RAD Experion machine using the RNA stdSens kit. All samples had an RNA-quality index (RQI) above 8.

RNA-seq library construction was performed by purifying the mRNA by oligo(dT)beads, the resulting library was sequenced on an Illumina HiSeq 2000 sequencing platform with TruSeqV3 sequencing reagents at the Beijing Genomics Institute, Shenzhen, PR China as previously described^46^.

### Differential expression analysis

Quality control for total RNA-Seq reads was performed using FastQC software. Reads were aligned to the pig reference genome (Sscrofa 11.1) using the Bioconductor Rsubread package^47^ with default parameters. Genes were annotated using the Ensembl database^48^ and filtered excluding genes which were expressed in less than twenty percent of the animals. The same phenotype as for the GWAS (RFI) was used as a response variable in the DE-analysis, i.e., the animals were divided into high and low RFI groups using 0 MJ NE per day as a threshold. As mentioned above, pigs for RNA sequencing were initially selected based on FCR values adjusted to the body weight at the start of the trial. However, high and low FE grouping based on FCR and RFI matched for all pigs. Mean values of RFI for the low and high RFI groups were -1.56 and 1.56, respectively, standard deviation were 0.57 and 0.88, respectively. Analysis of differential expression was carried out using DESeq2 platform V.1.36^49^. After the first fit of the model, Cook’s distances for gene expression were retrieved using the assays () function in DESeq2 in order to detect outliers. In this way one animal was considered as an outlier based on average Cook’s distance value (Suppl. Figure 4). This pig was excluded from further analysis and the final DE model was fitted using the remaining dataset. Features with a False Discovery Rate (FDR) ≤  0.1 were considered to be differentially expressed genes (DE-genes).

Functional enrichment analysis for the full result table of DE association test was carried out using the STRING V. 11.5 web-tool (www.string-db.org)^50^ based on Kolmogorov–Smirnov test statistics. Functional enrichment was run on two estimates of DE analysis, namely nominal* p-*values and log fold changes for genes.

### Cis-eQTL analysis

For cis-eQTL analysis, transcriptome and genotype data for 94 out of the 96 pigs with transcriptome data were used. One pig was excluded after DE-analysis as described above and another pig lacked genotype data. The RNA-seq data were mapped and filtered as for the DE-analysis and the data was normalized by size factor using the DESeq2 platform V.1.36^49^. Quality control of SNPs was conducted as for GWAS. Cis-QTL were mapped using the BootstrapQTL method V 1.0.5^21^. BootstrapQTL uses three steps of multiple testing correction. At first Bonferroni correction is used to adjust nominal* p-*values from MatrixEQTL cis-SNPs separately for each gene, after that FDR correction is applied to the lowest* p-*values for all genes, and in the end SNPs with locally adjusted* p-*values corresponding to globally corrected* p-*value threshold of 0.05 are considered as eSNPs. The bootstrap procedure was used to correct for the overestimation of effect sizes. Cis-eQTL associations were searched within a 2 Mb region around each gene.

### SNP calling using RNA-seq data

For SNP calling, increased requirements for reads were applied. Read head cutting (12 bases) and sliding window trimming (SLIDINGWINDOW:4:20) were applied for all reads using Trimmomatic^51^. Then reads were aligned to the pig reference genome (Sscrofa 11.1) using the STAR package V.2.7.10^52^ with default parameters. SNP calling was performed using the Rsubread package V.2.0.3^47^. Only polymorphisms with a score higher than 0.2 were kept. The identified polymorphisms were annotated using Ensembl Variant Effect Predictor tool^22^.

### Transcription factor regulation

The GeneHancer database V.5.9^53^ was used to build transcription factor regulatory networks. The database was filtered to include only promoters and promoter genes with “double elite” confidence scores^53^. Genes located in QTL regions were tested as transcription factors regulating the expression of DE-genes. If the target DE gene was a transcription factor, its potential role in regulating expression of other DE-genes was also tested. In this way, a network was built starting with the QTL transcription factor coding gene and ending with all targets found.

## Supplementary Information

Supplementary Information 1.

## Data Availability

The datasets generated and analyzed during the current study are available from the Genome Sequence Archive (GSA) repository with the primary accession code CRA007239.
