# Supplementary material for: Analysis of merged transcriptomic and genomic datasets to identify genes and pathways underlying residual feed intake in growing pigs
Source: Sci Rep. 2022 Dec 19;12:21946. doi: 10.1038/s41598-022-26496-1 (PMC9763391; doi:10.1038/s41598-022-26496-1)
Supplement: Supplementary file 1 — Supplementary Information 1. [file 41598_2022_26496_MOESM1_ESM.pdf]

Supplemental Information

Analysis of merged transcriptomic and genomic datasets to identify genes and pathways underlying residual feed intake in growing pigs

Emil Ibragimov, Anni Øyan Pedersen, Liang Xiao, Susanna Cirera, Merete Fredholm, Peter Karlskov-Mortensen

Supplemental Figure 1

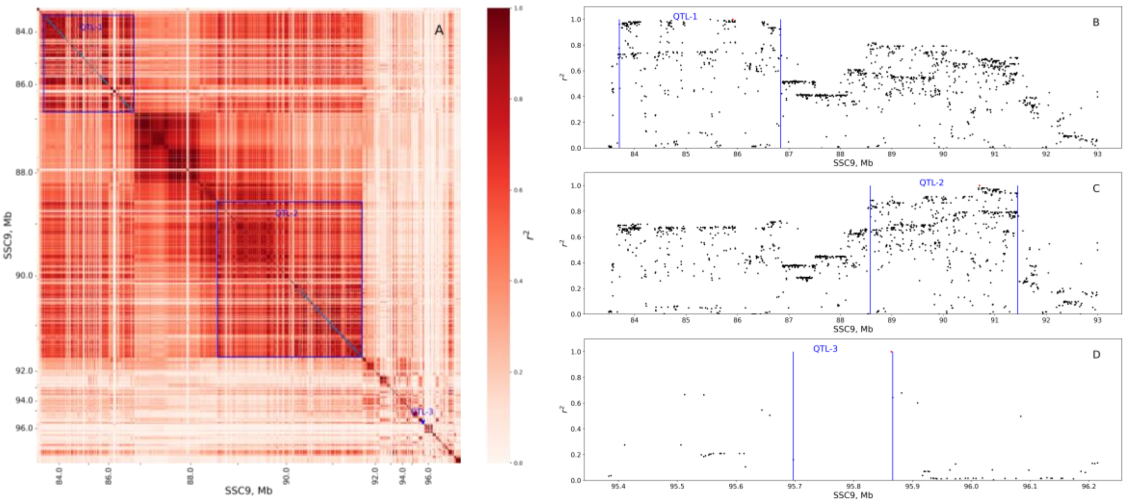

Suppl. Fig.1 – LD for SNPs significantly associated with RFI. A – heatmap of r^2 is shown. Blue dots indicate positions of genome-wide significant SNPs, blue lines indicate borders of QTL regions; B,C,D –LD between lead SNP in region (red dot) and surrounding SNPs.

Supplemental Figure 2

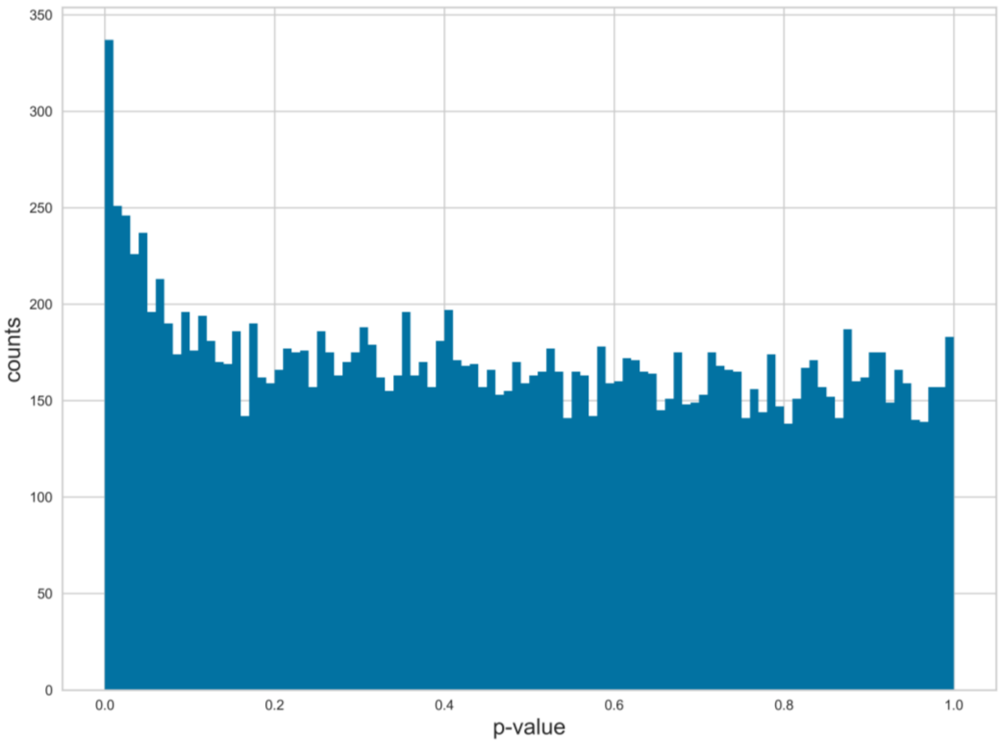

Suppl. Fig. 2 – Histogram of p-value for the DE analysis for RFI

Supplemental Figure 3

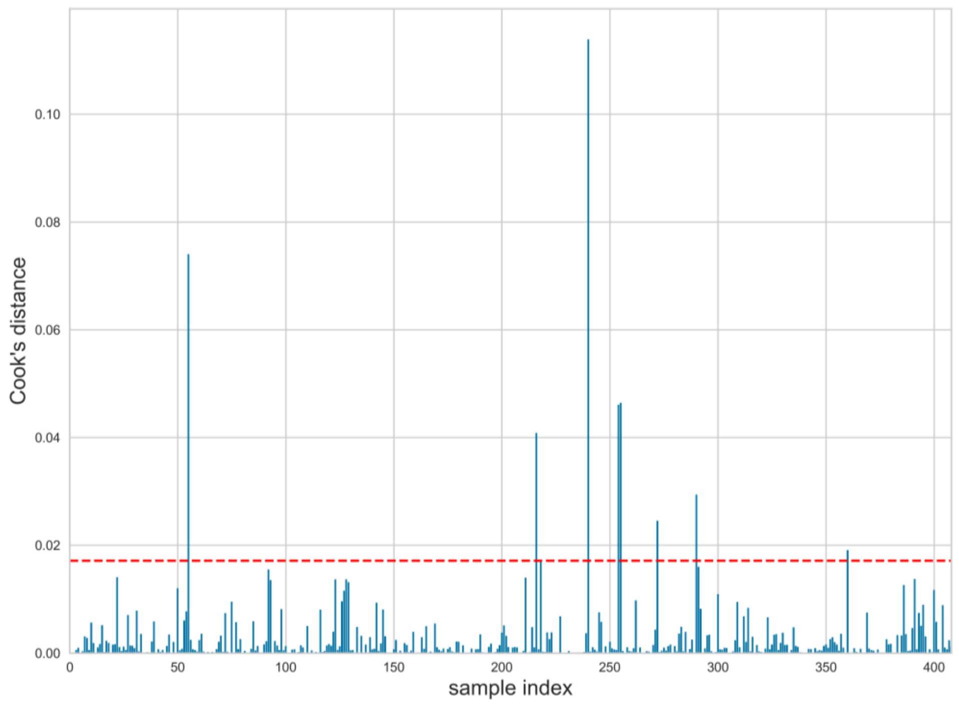

Suppl. Fig. 3 – The phenotype outlier detection. Cook's distances were calculated using the multiple regression model for expected ADFI. The dashed red line indicates the threshold value (the 98th percentile).

Supplemental Figure 4

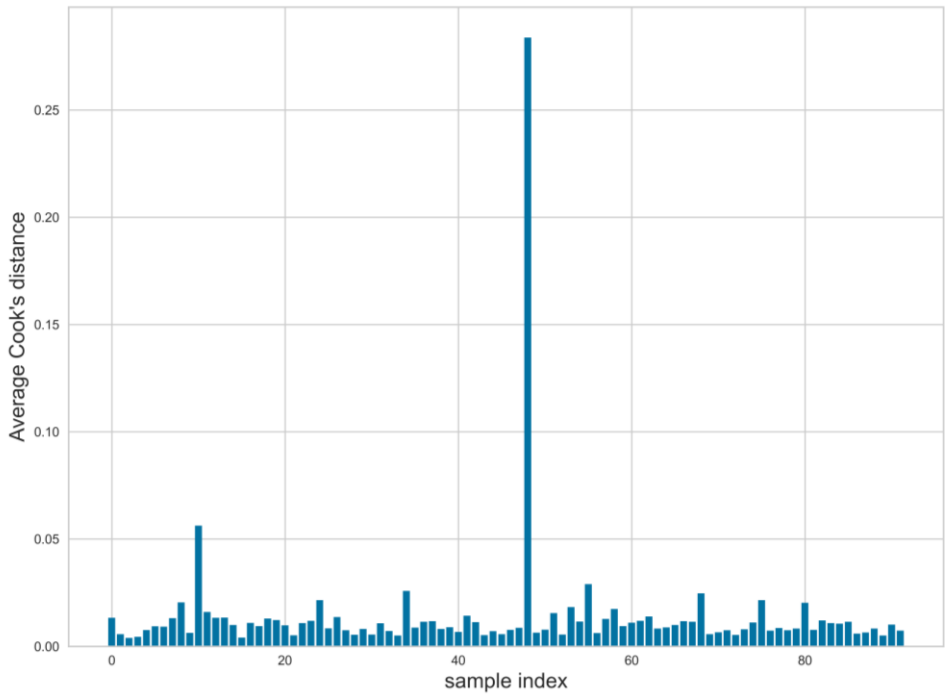

Suppl. Fig. 4 – Gene expression outlier detection. For each sample an average Cook's distance based on all genes included in DE-analysis was calculated. The sample with the highest value was excluded from further analyses.

Suppl. Table 1: Genes located in QTLs.

Gene name	Chromosome	Left bp	Right bp	Ensembl biotype	QTL
DGKB	9	83569920	84510087	protein_coding	QTL-1
AGMO	9	84470277	84894843	protein_coding	QTL-1
U2	9	84738014	84738145	snRNA	QTL-1
ENSSSCG00000042517	9	84754816	84759401	pseudogene	QTL-1
ENSSSCG00000043100	9	84864192	84867029	protein_coding	QTL-1
MEOX2	9	84943197	85010157	protein_coding	QTL-1
ENSSSCG00000046863	9	85011895	85013422	lncRNA	QTL-1
ENSSSCG00000041560	9	85012018	85020122	lncRNA	QTL-1
ENSSSCG00000048170	9	85023913	85029881	protein_coding	QTL-1
CRPPA	9	85415550	85710752	protein_coding	QTL-1
ENSSSCG00000051224	9	85495576	85497634	pseudogene	QTL-1
ENSSSCG00000032864	9	85566098	85567136	protein_coding	QTL-1
SOSTDC1	9	85743335	85747591	protein_coding	QTL-1
LRRC72	9	85803124	85844803	protein_coding	QTL-1
ANKMY2	9	85848002	85887427	protein_coding	QTL-1
BZW2	9	85886158	85947408	protein_coding	QTL-1
TSPAN13	9	85996640	86033354	protein_coding	QTL-1
AGR2	9	86039015	86053857	protein_coding	QTL-1
ENSSSCG00000046663	9	86262425	86268978	lncRNA	QTL-1
ENSSSCG00000048277	9	86337636	86340325	protein_coding	QTL-1
ENSSSCG00000044725	9	86487601	86511181	lncRNA	QTL-1
AHR	9	86511369	86555943	protein_coding	QTL-1
ENSSSCG00000044052	9	86634902	86642522	protein_coding	QTL-1
ENSSSCG00000046719	9	86782583	86801932	lncRNA	QTL-1
ENSSSCG00000044171	9	88623752	88631633	lncRNA	QTL-2
ENSSSCG00000042617	9	88739064	88742446	lncRNA	QTL-2
POLR1F	9	88760398	88785802	protein_coding	QTL-2
ENSSSCG00000046636	9	88774011	88778275	pseudogene	QTL-2
ENSSSCG00000048521	9	88794910	88796687	lncRNA	QTL-2
TMEM196	9	88798827	88858013	protein_coding	QTL-2
MACC1	9	89102621	89210309	protein_coding	QTL-2
ITGB8	9	89341141	89450679	protein_coding	QTL-2
ENSSSCG00000049419	9	89409912	89412059	processed_pseudogene	QTL-2
ABCB5	9	89595284	89738368	protein_coding	QTL-2
SP8	9	89764764	89767425	protein_coding	QTL-2
ENSSSCG00000045940	9	89887473	89889927	processed_pseudogene	QTL-2
SP4	9	90336690	90410295	protein_coding	QTL-2
DNAH11	9	90423077	90767487	protein_coding	QTL-2
ENSSSCG00000042690	9	90440588	90445294	protein_coding	QTL-2
ENSSSCG00000043741	9	90654785	90658603	protein_coding	QTL-2
CDCA7L	9	90766767	90806865	protein_coding	QTL-2
ENSSSCG00000035257	9	90950852	90951232	protein_coding	QTL-2
RAPGEF5	9	90958078	91578992	protein_coding	QTL-2

Suppl. Table 2: Differentially Expressed genes.

gene_name	baseMean	log2FoldChange	lfcSE	stat	pvalue	FDR
MARCO	15.71	2.073	0.424	4.894	9.87E-07	0.0084
TRAV16	6.91	1.871	0.376	4.978	6.44E-07	0.0084
ENSSSCG00000046518	2180.72	2.566	0.558	4.596	4.31E-06	0.0203
CXCL11	114.64	1.555	0.34	4.576	4.75E-06	0.0203
CD5	636.74	0.626	0.143	4.366	1.26E-05	0.0432
ENSSSCG00000025271	193.66	0.942	0.219	4.296	1.74E-05	0.0495
PPP3CA	689.48	0.367	0.087	4.232	2.32E-05	0.0544
CALHM6	121.18	0.81	0.194	4.184	2.87E-05	0.0544
SNX10	282.99	0.798	0.19	4.204	2.63E-05	0.0544
ENSSSCG00000034614	218.04	-0.322	0.078	-4.113	3.91E-05	0.0651
GNG10	642.95	0.435	0.106	4.097	4.19E-05	0.0651
IFNAR1	1222.54	0.276	0.069	4.027	5.64E-05	0.0803
SRGN	1645.41	0.663	0.165	4.007	6.15E-05	0.0809
ENSSSCG00000022947	829.05	-0.254	0.064	-3.947	7.93E-05	0.0903
IRF2	927.43	0.265	0.067	3.963	7.41E-05	0.0903
LXN	361	0.334	0.085	3.922	8.77E-05	0.0937
ENSSSCG00000033382	31.19	2.975	0.764	3.893	9.91E-05	0.0996

Suppl. Table 3: Rank-based enrichment test for DE analysis results (based on p-values).

category	term ID	term description	genes mapped	enrichment score	FDR
GO Process	GO:0050863	Regulation of t cell activation	160	0.853926	1.86E-07
GO Process	GO:1903037	Regulation of leukocyte cell-cell adhesion	149	0.881867	1.86E-07
GO Process	GO:0002696	Positive regulation of leukocyte activation	154	0.726468	1.5E-06
GO Process	GO:0050867	Positive regulation of cell activation	161	0.70816	1.52E-06
GO Process	GO:1903039	Positive regulation of leukocyte cell-cell adhesion	110	0.896731	1.98E-06
GO Process	GO:0051251	Positive regulation of lymphocyte activation	135	0.770889	2.59E-06
GO Process	GO:0050870	Positive regulation of t cell activation	97	0.910962	1.12E-05
GO Process	GO:0045619	Regulation of lymphocyte differentiation	104	0.826927	1.86E-05
GO Process	GO:0022409	Positive regulation of cell-cell adhesion	131	0.703475	0.00031
GO Process	GO:0070663	Regulation of leukocyte proliferation	106	0.848784	0.00035
GO Process	GO:0050670	Regulation of lymphocyte proliferation	105	0.829606	0.00065
GO Process	GO:0045580	Regulation of t cell differentiation	84	0.762499	0.0017
GO Process	GO:1902105	Regulation of leukocyte differentiation	144	0.603402	0.0018
GO Process	GO:0042129	Regulation of t cell proliferation	78	0.93524	0.002

GO Process	GO:0042102	Positive regulation of t cell proliferation	46	1.09379	0.0026
GO Process	GO:0007159	Leukocyte cell-cell adhesion	26	1.08023	0.0033
GO Process	GO:0048002	Antigen processing and presentation of peptide antigen	29	1.87474	0.0048
GO Process	GO:0060326	Cell chemotaxis	105	0.730724	0.0048
GO Process	GO:0002703	Regulation of leukocyte mediated immunity	104	0.585696	0.0085
GO Process	GO:0050900	Leukocyte migration	102	0.68836	0.009
GO Component	GO:0009897	External side of plasma membrane	157	0.771634	0.00084
STRING clusters	CL:726	Mixed, incl. allograft rejection, and t cell receptor complex	25	1.92018	0.00011
STRING clusters	CL:1456	Mixed, incl. chemokine-mediated signaling pathway, and protein complex involved in cell adhesion	69	1.13067	0.00034
STRING clusters	CL:19423	Cytosolic ribosome	56	0.277707	0.0019
STRING clusters	CL:19430	Ribosome, and cytosolic small ribosomal subunit	50	0.086305	0.0044
STRING clusters	CL:2139	Mixed, incl. antigen processing and presentation of peptide antigen via mhc class i, and immunoregulatory interactions between a lymphoid and a non-lymphoid cell	60	1.26259	0.0044
STRING clusters	CL:19428	Cytosolic ribosome	53	0.275033	0.0047
STRING clusters	CL:2140	Mixed, incl. antigen processing and presentation of peptide antigen via mhc class i, and immunoregulatory interactions between a lymphoid and a non-lymphoid cell	56	1.32529	0.0049
STRING clusters	CL:2141	Mixed, incl. antigen processing and presentation of peptide antigen via mhc class i, and immunoregulatory interactions between a lymphoid and a non-lymphoid cell	37	1.5148	0.0049
KEGG	ssc04612	Antigen processing and presentation	39	1.78151	0.00085
KEGG	ssc05320	Autoimmune thyroid disease	23	1.91636	0.0039
KEGG	ssc05340	Primary immunodeficiency	21	1.58394	0.0039
KEGG	ssc04060	Cytokine-cytokine receptor interaction	134	0.57398	0.0045
KEGG	ssc04940	Type I diabetes mellitus	26	1.52273	0.0045
KEGG	ssc05144	Malaria	32	0.85202	0.0045

KEGG	ssc05140	Leishmaniasis	43	1.3064	0.0057
Pfam	PF07654	Immunoglobulin C1-set domain	33	1.82296	0.0017
InterPro	IPR003597	Immunoglobulin C1-set	26	2.09077	0.0041
InterPro	IPR003006	Immunoglobulin/major histocompatibility complex, conserved site	23	2.23977	0.0043
SMART	SM00407	Immunoglobulin C-Type	28	2.08806	0.0004

Suppl. Table 4: Rank-based enrichment test results for DE analysis results (based on log fold change values). Values were ranked in ascending order, thus top and bottom directions indicate downregulation and upregulation in the high RFI group of pigs.

category	term ID	term description	genes mapped	enrichment score	direction	FDR
GO Process	GO:0002250	Adaptive immune response	163	0.601892	bottom	7.7E-12
GO Process	GO:0050867	Positive regulation of cell activation	162	0.586475	bottom	1.92E-11
GO Process	GO:0002696	Positive regulation of leukocyte activation	155	0.583127	bottom	9.8E-11
GO Process	GO:0051251	Positive regulation of lymphocyte activation	135	0.640014	bottom	2.96E-10
GO Process	GO:1903037	Regulation of leukocyte cell-cell adhesion	149	0.551598	bottom	6.44E-10
GO Process	GO:1903039	Positive regulation of leukocyte cell-cell adhesion	110	0.626833	bottom	8.96E-10
GO Process	GO:0050870	Positive regulation of t cell activation	97	0.684668	bottom	1.64E-09
GO Process	GO:0050863	Regulation of t cell activation	160	0.517244	bottom	2.81E-09
GO Process	GO:0019221	Cytokine-mediated signaling pathway	178	0.490811	bottom	2.86E-09
GO Process	GO:0070663	Regulation of leukocyte proliferation	106	0.632395	bottom	4.82E-09
GO Process	GO:0050670	Regulation of lymphocyte proliferation	105	0.624004	bottom	1.07E-08
GO Process	GO:0022409	Positive regulation of cell-cell adhesion	131	0.549031	bottom	2.11E-08
GO Process	GO:0050900	Leukocyte migration	102	0.671974	bottom	9.44E-08
GO Process	GO:0001819	Positive regulation of cytokine production	200	0.471619	bottom	1.42E-07
GO Process	GO:0042102	Positive regulation of t cell proliferation	46	0.81245	bottom	1.51E-07
GO Process	GO:0060326	Cell chemotaxis	105	0.723738	bottom	1.51E-07
GO Process	GO:0050671	Positive regulation of lymphocyte proliferation	64	0.757587	bottom	2.19E-07
GO Process	GO:0070665	Positive regulation of leukocyte proliferation	65	0.750836	bottom	3.9E-07
GO Process	GO:0050727	Regulation of inflammatory response	167	0.450219	bottom	4.79E-06
GO Process	GO:0042129	Regulation of t cell proliferation	78	0.574171	bottom	4.86E-06

GO Process	GO:0030595	Leukocyte chemotaxis	62	0.905681	bottom	6.15E-06
GO Process	GO:0007159	Leukocyte cell-cell adhesion	26	0.922209	bottom	8.78E-06
GO Process	GO:0045619	Regulation of lymphocyte differentiation	104	0.455794	bottom	1.13E-05
GO Process	GO:1902107	Positive regulation of leukocyte differentiation	77	0.575899	bottom	2.22E-05
GO Process	GO:0002695	Negative regulation of leukocyte activation	88	0.590644	bottom	0.000023
GO Process	GO:0050864	Regulation of b cell activation	66	0.606999	bottom	0.000023
GO Process	GO:1902105	Regulation of leukocyte differentiation	144	0.416607	bottom	0.000023
GO Process	GO:1990266	Neutrophil migration	35	1.09513	bottom	2.33E-05
GO Process	GO:0071219	Cellular response to molecule of bacterial origin	92	0.669449	bottom	2.47E-05
GO Process	GO:0002237	Response to molecule of bacterial origin	135	0.496749	bottom	2.59E-05
GO Process	GO:0007059	Chromosome segregation	178	0.192857	top	3.01E-05
GO Process	GO:0042110	T cell activation	126	0.392008	bottom	3.42E-05
GO Process	GO:0071216	Cellular response to biotic stimulus	107	0.623586	bottom	3.42E-05
GO Process	GO:0030593	Neutrophil chemotaxis	30	1.21982	bottom	3.92E-05
GO Process	GO:0002699	Positive regulation of immune effector process	112	0.444282	bottom	3.93E-05
GO Process	GO:0071222	Cellular response to lipopolysaccharide	84	0.708097	bottom	4.52E-05
GO Process	GO:0045621	Positive regulation of lymphocyte differentiation	57	0.637595	bottom	7.05E-05
GO Process	GO:0050866	Negative regulation of cell activation	99	0.535127	bottom	9.88E-05
GO Process	GO:0032103	Positive regulation of response to external stimulus	185	0.363496	bottom	0.00011
GO Process	GO:0097529	Myeloid leukocyte migration	49	0.921258	bottom	0.00011
GO Process	GO:0140014	Mitotic nuclear division	99	0.214775	top	0.00011
GO Process	GO:0032496	Response to lipopolysaccharide	126	0.498446	bottom	0.00012
GO Process	GO:0032663	Regulation of interleukin-2 production	30	0.664791	bottom	0.00012
GO Process	GO:0006814	Sodium ion transport	111	0.35415	both ends	0.00014
GO Process	GO:0031349	Positive regulation of defense response	122	0.447734	bottom	0.00014
GO Process	GO:0072676	Lymphocyte migration	31	0.971213	bottom	0.00014
GO Process	GO:0002822	Regulation of adaptive immune response based on somatic recombination of immune receptors built from	78	0.488645	bottom	0.00022

		immunoglobulin superfamily domains				
GO Process	GO:0048285	Organelle fission	190	0.21071	top	0.00023
GO Process	GO:0051250	Negative regulation of lymphocyte activation	71	0.572184	bottom	0.00024
GO Process	GO:0000280	Nuclear division	169	0.236366	top	0.00025
GO Process	GO:0045582	Positive regulation of t cell differentiation	47	0.713146	bottom	0.00025
GO Process	GO:0097530	Granulocyte migration	41	0.947666	bottom	0.00026
GO Process	GO:0006813	Potassium ion transport	86	0.32827	both ends	0.00031
GO Process	GO:0002697	Regulation of immune effector process	193	0.353304	bottom	0.00032
GO Process	GO:0034341	Response to interferon-gamma	47	0.768777	bottom	0.00033
GO Process	GO:0000819	Sister chromatid segregation	93	0.205958	top	0.00035
GO Process	GO:0071805	Potassium ion transmembrane transport	82	0.328045	both ends	0.00045
GO Process	GO:0002824	Positive regulation of adaptive immune response based on somatic recombination of immune receptors built from immunoglobulin superfamily domains	54	0.555638	bottom	0.00046
GO Process	GO:0071621	Granulocyte chemotaxis	35	1.06204	bottom	0.00047
GO Process	GO:1903708	Positive regulation of hemopoiesis	101	0.439201	bottom	0.00047
GO Process	GO:0002819	Regulation of adaptive immune response	84	0.466236	bottom	0.00053
GO Process	GO:0002703	Regulation of leukocyte mediated immunity	104	0.346452	bottom	0.00054
GO Process	GO:0070098	Chemokine-mediated signaling pathway	40	0.749994	bottom	0.00055
GO Process	GO:0098813	Nuclear chromosome segregation	132	0.217341	top	0.00057
GO Process	GO:0071346	Cellular response to interferon-gamma	37	0.944016	bottom	0.00065
GO Process	GO:1901617	Organic hydroxy compound biosynthetic process	86	0.426883	top	0.00067
GO Process	GO:0046165	Alcohol biosynthetic process	64	0.53918	top	0.00071
GO Process	GO:0045580	Regulation of t cell differentiation	84	0.42627	bottom	0.00076
GO Process	GO:1990869	Cellular response to chemokine	48	0.649667	bottom	0.00077
GO Process	GO:0007187	G protein-coupled receptor signaling pathway, coupled to cyclic nucleotide second messenger	104	0.283911	both ends	0.00079
GO Process	GO:0048002	Antigen processing and presentation of peptide antigen	29	0.49026	both ends	0.0008

GO Process	GO:0002521	Leukocyte differentiation	180	0.344865	bottom	0.00097
GO Process	GO:0002821	Positive regulation of adaptive immune response	56	0.533683	bottom	0.00097
GO Process	GO:0002706	Regulation of lymphocyte mediated immunity	71	0.422526	bottom	0.0011
GO Process	GO:0035725	Sodium ion transmembrane transport	94	0.336085	both ends	0.0011
GO Process	GO:0046634	Regulation of alpha-beta t cell activation	49	0.655585	bottom	0.0011
GO Process	GO:0048193	Golgi vesicle transport	177	0.182223	top	0.0011
GO Process	GO:0007218	Neuropeptide signaling pathway	32	0.555892	both ends	0.0012
GO Process	GO:0019882	Antigen processing and presentation	42	0.419197	both ends	0.0012
GO Process	GO:0042391	Regulation of membrane potential	169	0.246024	both ends	0.0012
GO Process	GO:0031343	Positive regulation of cell killing	31	0.877384	bottom	0.0013
GO Process	GO:0032649	Regulation of interferon-gamma production	47	0.553705	bottom	0.0013
GO Process	GO:0032729	Positive regulation of interferon-gamma production	36	0.64111	bottom	0.0013
GO Process	GO:0007188	Adenylate cyclase-modulating g protein-coupled receptor signaling pathway	97	0.284947	both ends	0.0014
GO Process	GO:0002274	Myeloid leukocyte activation	78	0.369773	bottom	0.0016
GO Process	GO:0002460	Adaptive immune response based on somatic recombination of immune receptors built from immunoglobulin superfamily domains	77	0.51679	bottom	0.0019
GO Process	GO:0002705	Positive regulation of leukocyte mediated immunity	73	0.359913	bottom	0.0019
GO Process	GO:0002831	Regulation of response to biotic stimulus	142	0.33663	bottom	0.0019
GO Process	GO:0000070	Mitotic sister chromatid segregation	73	0.211103	top	0.002
GO Process	GO:0006695	Cholesterol biosynthetic process	22	0.937129	top	0.0023
GO Process	GO:0016126	Sterol biosynthetic process	26	0.911109	top	0.0023
GO Process	GO:1902653	Secondary alcohol biosynthetic process	24	0.928624	top	0.0023
GO Process	GO:0002253	Activation of immune response	123	0.387696	bottom	0.0024
GO Process	GO:0002443	Leukocyte mediated immunity	98	0.342588	bottom	0.0025
GO Process	GO:0070646	Protein modification by small protein removal	90	0.16414	top	0.0026

GO Process	GO:2000106	Regulation of leukocyte apoptotic process	39	0.709092	bottom	0.0028
GO Process	GO:0031341	Regulation of cell killing	38	0.694578	bottom	0.0034
GO Process	GO:0008015	Blood circulation	138	0.201487	both ends	0.0035
GO Process	GO:0002708	Positive regulation of lymphocyte mediated immunity	52	0.432677	bottom	0.0036
GO Process	GO:0019932	Second-messenger-mediated signaling	176	0.209697	both ends	0.0036
GO Process	GO:0071103	DNA conformation change	159	0.205706	top	0.0039
GO Process	GO:0042742	Defense response to bacterium	64	0.505318	bottom	0.0042
GO Process	GO:0006302	Double-strand break repair	120	0.234301	top	0.0043
GO Process	GO:0016579	Protein deubiquitination	79	0.171527	top	0.0043
GO Process	GO:0034765	Regulation of ion transmembrane transport	198	0.226924	both ends	0.0043
GO Process	GO:0050871	Positive regulation of b cell activation	42	0.603227	bottom	0.0048
GO Process	GO:0046635	Positive regulation of alpha-beta t cell activation	31	0.740098	bottom	0.005
GO Process	GO:0050729	Positive regulation of inflammatory response	65	0.611829	bottom	0.005
GO Process	GO:0031663	Lipopolysaccharide-mediated signaling pathway	24	0.773421	bottom	0.0051
GO Process	GO:0006066	Alcohol metabolic process	173	0.302185	top	0.0052
GO Process	GO:0045088	Regulation of innate immune response	83	0.371454	bottom	0.0053
GO Process	GO:0030888	Regulation of b cell proliferation	32	0.745787	bottom	0.0059
GO Process	GO:0007600	Sensory perception	182	0.210647	both ends	0.006
GO Process	GO:0018022	Peptidyl-lysine methylation	56	0.304096	top	0.0075
GO Process	GO:0032652	Regulation of interleukin-1 production	29	0.673098	bottom	0.0075
GO Process	GO:0032653	Regulation of interleukin-10 production	23	0.849537	bottom	0.0077
GO Process	GO:0002263	Cell activation involved in immune response	100	0.344856	bottom	0.0079
GO Process	GO:0002700	Regulation of production of molecular mediator of immune response	72	0.452566	bottom	0.0082
GO Process	GO:0002764	Immune response-regulating signaling pathway	86	0.388774	bottom	0.0084
GO Process	GO:0003341	Cilium movement	65	0.377303	both ends	0.0087
GO Process	GO:1903555	Regulation of tumor necrosis factor superfamily cytokine production	67	0.556883	bottom	0.0087

GO Process	GO:0071356	Cellular response to tumor necrosis factor	80	0.493817	bottom	0.0093
GO Process	GO:0035637	Multicellular organismal signaling	45	0.385822	both ends	0.0095
GO Process	GO:0070661	Leukocyte proliferation	44	0.586947	bottom	0.0096
GO Process	GO:0032732	Positive regulation of interleukin-1 production	21	0.783435	bottom	0.0098
GO Process	GO:0034612	Response to tumor necrosis factor	92	0.440678	bottom	0.0099
GO Function	GO:0140375	Immune receptor activity	82	0.752268	bottom	2.23E-07
GO Function	GO:0004930	G protein-coupled receptor activity	181	0.338368	both ends	2.93E-07
GO Function	GO:0003735	Structural constituent of ribosome	114	0.342122	bottom	3.33E-07
GO Function	GO:0019955	Cytokine binding	82	0.571234	bottom	6.16E-07
GO Function	GO:0022836	Gated channel activity	148	0.363522	both ends	8.43E-07
GO Function	GO:0005261	Cation channel activity	157	0.358159	both ends	9.5E-07
GO Function	GO:0048018	Receptor ligand activity	196	0.37191	both ends	3.36E-06
GO Function	GO:0004896	Cytokine receptor activity	69	0.709071	bottom	6.86E-06
GO Function	GO:0005125	Cytokine activity	93	0.473248	bottom	5.79E-05
GO Function	GO:0001653	Peptide receptor activity	63	0.439916	both ends	9.26E-05
GO Function	GO:0004386	Helicase activity	120	0.295868	top	9.26E-05
GO Function	GO:0008528	G protein-coupled peptide receptor activity	59	0.435678	bottom	0.00044
GO Function	GO:0005126	Cytokine receptor binding	110	0.419592	bottom	0.00063
GO Function	GO:0016874	Ligase activity	120	0.256905	top	0.00072
GO Function	GO:0042379	Chemokine receptor binding	24	0.999113	bottom	0.0012
GO Function	GO:0005244	Voltage-gated ion channel activity	85	0.362769	both ends	0.0014
GO Function	GO:0005518	Collagen binding	48	0.568149	bottom	0.0014
GO Function	GO:0015276	Ligand-gated ion channel activity	49	0.466934	both ends	0.0014
GO Function	GO:0001664	G protein-coupled receptor binding	131	0.293124	bottom	0.0019
GO Function	GO:0005267	Potassium channel activity	50	0.403609	both ends	0.0022
GO Function	GO:0015081	Sodium ion transmembrane transporter activity	90	0.339436	both ends	0.0043
GO Function	GO:0004843	Thiol-dependent ubiquitin-specific protease activity	71	0.190027	top	0.0045
GO Function	GO:0005230	Extracellular ligand-gated ion channel activity	24	0.651527	both ends	0.0052
GO Function	GO:0015079	Potassium ion transmembrane transporter activity	74	0.286493	both ends	0.0052
GO Function	GO:0005044	Scavenger receptor activity	30	1.07714	bottom	0.0053

GO Function	GO:0005249	Voltage-gated potassium channel activity	32	0.537637	both ends	0.0053
GO Function	GO:0008094	DNA-dependent ATPase activity	72	0.361827	top	0.0053
GO Function	GO:0008242	Omega peptidase activity	75	0.207333	top	0.006
GO Function	GO:0099094	Ligand-gated cation channel activity	39	0.483137	both ends	0.006
GO Function	GO:0022843	Voltage-gated cation channel activity	53	0.465945	both ends	0.0068
GO Function	GO:0003774	Motor activity	87	0.295205	top	0.007
GO Function	GO:0015370	Solute:sodium symporter activity	42	0.484164	both ends	0.007
GO Function	GO:0030594	Neurotransmitter receptor activity	30	0.582718	both ends	0.0074
GO Function	GO:0003823	Antigen binding	25	0.874966	bottom	0.0084
GO Component	GO:0022626	Cytosolic ribosome	67	0.627752	bottom	1.69E-17
GO Component	GO:0009897	External side of plasma membrane	157	0.611329	bottom	4.65E-15
GO Component	GO:0022627	Cytosolic small ribosomal subunit	33	0.641169	bottom	5.72E-09
GO Component	GO:0022625	Cytosolic large ribosomal subunit	33	0.664503	bottom	1.47E-07
GO Component	GO:0044391	Ribosomal subunit	123	0.312691	bottom	8.75E-07
GO Component	GO:0005840	Ribosome	144	0.263493	bottom	4.22E-06
GO Component	GO:1902495	Transmembrane transporter complex	131	0.352318	both ends	5.35E-05
GO Component	GO:0034702	Ion channel complex	124	0.365643	both ends	6.63E-05
GO Component	GO:1990351	Transporter complex	136	0.319375	both ends	0.00022
GO Component	GO:0015935	Small ribosomal subunit	50	0.368989	bottom	0.00032
GO Component	GO:0034705	Potassium channel complex	31	0.545979	both ends	0.00041
GO Component	GO:0098562	Cytoplasmic side of membrane	111	0.275701	bottom	0.00067
GO Component	GO:0034703	Cation channel complex	94	0.377026	both ends	0.0011
GO Component	GO:0000793	Condensed chromosome	132	0.224321	top	0.0018
GO Component	GO:0031225	Anchored component of membrane	76	0.365477	bottom	0.0018
GO Component	GO:0015934	Large ribosomal subunit	74	0.273283	bottom	0.0048
GO Component	GO:0098802	Plasma membrane signaling receptor complex	90	0.365255	bottom	0.0048
STRING clusters	CL:19418	Cytosolic ribosome	58	0.690085	bottom	2E-20
STRING clusters	CL:19423	Cytosolic ribosome	56	0.721436	bottom	2E-20
STRING clusters	CL:19404	Cytosolic ribosome	62	0.637683	bottom	2.75E-20
STRING clusters	CL:19413	Cytosolic ribosome	59	0.651986	bottom	4.63E-20
STRING clusters	CL:19366	Cytosolic ribosome, and ribosomal protein l44	73	0.559774	bottom	1.58E-19
STRING clusters	CL:19428	Cytosolic ribosome	53	0.724749	bottom	1.58E-19
STRING clusters	CL:19430	Ribosome, and cytosolic small ribosomal subunit	50	0.736591	bottom	2.41E-19

STRING clusters	CL:19370	Cytosolic ribosome	72	0.560669	bottom	3.58E-19
STRING clusters	CL:19399	Cytosolic ribosome	67	0.594907	bottom	7.15E-19
STRING clusters	CL:19374	Cytosolic ribosome	71	0.543734	bottom	8.62E-19
STRING clusters	CL:19394	Cytosolic ribosome	68	0.572925	bottom	2.07E-18
STRING clusters	CL:19389	Cytosolic ribosome	70	0.538341	bottom	2.16E-18
STRING clusters	CL:1456	Mixed, incl. chemokine-mediated signaling pathway, and protein complex involved in cell adhesion	69	1.00944	bottom	2.91E-17
STRING clusters	CL:19364	Cytosolic ribosome, and ribosomal protein l44	79	0.494095	bottom	6.23E-17
STRING clusters	CL:19357	Cytosolic ribosome, and translation elongation factor activity	85	0.483637	bottom	3.93E-16
STRING clusters	CL:19360	Cytosolic ribosome, and translation elongation factor activity	81	0.486015	bottom	4.15E-16
STRING clusters	CL:19432	Ribosome	40	0.682082	bottom	6.64E-16
STRING clusters	CL:19440	Ribosome	36	0.664535	bottom	3.68E-15
STRING clusters	CL:19436	Ribosome	38	0.65855	bottom	6.12E-15
STRING clusters	CL:19445	Ribosome	34	0.658462	bottom	8.81E-14
STRING clusters	CL:19447	Ribosome	26	0.644053	bottom	7.71E-10
STRING clusters	CL:19449	Ribosome	21	0.655976	bottom	1.32E-08
STRING clusters	CL:1458	Chemokine-mediated signaling pathway	37	1.04275	bottom	2.25E-07
STRING clusters	CL:1591	Mixed, incl. leukocyte cell-cell adhesion, and lysosome-associated membrane glycoprotein (lamp)	31	0.953772	bottom	2.25E-07
STRING clusters	CL:1461	Chemokine receptors bind chemokines, and Chemokine interleukin-8-like domain	33	1.10202	bottom	2.29E-07
STRING clusters	CL:726	Mixed, incl. allograft rejection, and t cell receptor complex	25	1.21544	bottom	1.04E-06
STRING clusters	CL:12705	Mixed, incl. nucleotide receptor activity, and immunoglobulin	64	0.83394	bottom	4.29E-06
STRING clusters	CL:1464	Chemokine receptors bind chemokines, and CCR3 chemokine receptor binding	30	1.07566	bottom	6.15E-06
STRING clusters	CL:2139	Mixed, incl. antigen processing and presentation of peptide antigen via mhc class i, and immunoregulatory interactions between a	60	0.573509	bottom	2.35E-05

		lymphoid and a non-lymphoid cell				
STRING clusters	CL:2141	Mixed, incl. antigen processing and presentation of peptide antigen via mhc class i, and immunoregulatory interactions between a lymphoid and a non-lymphoid cell	37	0.634907	bottom	3.98E-05
STRING clusters	CL:508	Mixed, incl. sh2 domain, and asthma	98	0.509173	bottom	5.27E-05
STRING clusters	CL:2140	Mixed, incl. antigen processing and presentation of peptide antigen via mhc class i, and immunoregulatory interactions between a lymphoid and a non-lymphoid cell	56	0.548757	bottom	6.55E-05
STRING clusters	CL:12706	Mixed, incl. immunoglobulin like, and immunoregulatory interactions between a lymphoid and a non-lymphoid cell	34	1.17586	bottom	7.52E-05
STRING clusters	CL:506	Mixed, incl. sh2 domain, and transmembrane receptor protein tyrosine kinase activity	115	0.413542	bottom	0.00014
STRING clusters	CL:1466	Chemokine receptors bind chemokines, and CCR3 chemokine receptor binding	26	0.961409	bottom	0.00039
STRING clusters	CL:17383	Mixed, incl. condensed chromosome, centromeric region, and anaphase-promoting complex	99	0.24958	top	0.00043
STRING clusters	CL:17385	Mixed, incl. condensed chromosome, centromeric region, and microtubule binding	86	0.249361	top	0.00077
STRING clusters	CL:17387	Mixed, incl. mitotic sister chromatid segregation, and condensed chromosome outer kinetochore	62	0.272673	top	0.001
STRING clusters	CL:1592	Mixed, incl. leukocyte cell-cell adhesion, and intercellular adhesion molecule, n-terminal	23	0.844003	bottom	0.0011
STRING clusters	CL:1142	JAK-STAT signaling pathway, and cytokine receptor activity	77	0.471455	bottom	0.0012
STRING clusters	CL:1150	JAK-STAT signaling pathway, and receptor	53	0.499022	bottom	0.0015

		signaling pathway via STAT				
STRING clusters	CL:17386	Mixed, incl. condensed chromosome, centromeric region, and condensin complex	74	0.251778	top	0.0015
STRING clusters	CL:28545	Sterol biosynthetic process, and bile acid biosynthetic process	22	1.05461	top	0.0017
STRING clusters	CL:1686	Mixed, incl. toll-like receptor cascades, and tumor necrosis factor receptor binding	81	0.362062	bottom	0.0021
STRING clusters	CL:2142	Mixed, incl. antigen processing and presentation of peptide antigen via mhc class i, and natural killer cell receptor-like, c-type lectin-like domain	27	0.48349	bottom	0.0021
STRING clusters	CL:16746	DNA replication, and Fanconi anemia pathway	116	0.257149	top	0.0025
STRING clusters	CL:1685	Mixed, incl. toll-like receptor cascades, and tumor necrosis factor receptor binding	89	0.318902	bottom	0.0043
STRING clusters	CL:17389	Mixed, incl. mitotic sister chromatid segregation, and negative regulation of mitotic sister chromatid separation	53	0.272053	top	0.0049
STRING clusters	CL:1143	JAK-STAT signaling pathway, and receptor signaling pathway via STAT	63	0.482792	bottom	0.0051
STRING clusters	CL:637	Mixed, incl. fceri mediated ca+2 mobilization, and b cell receptor signaling pathway	30	0.64202	bottom	0.0055
STRING clusters	CL:20638	tRNA aminoacylation, and regulation of mitochondrial RNA catabolic process	61	0.202837	top	0.0066
STRING clusters	CL:1687	Mixed, incl. toll-like receptor cascades, and death domain	75	0.330889	bottom	0.0077
STRING clusters	CL:20076	rRNA processing, and tRNA methylation	130	0.162113	top	0.0077
STRING clusters	CL:1684	Mixed, incl. nf-kappa b signaling pathway, and positive regulation of cysteine-type endopeptidase activity	107	0.259675	bottom	0.0097
KEGG	ssc04060	Cytokine-cytokine receptor interaction	134	0.544192	bottom	8.69E-13
KEGG	ssc04080	Neuroactive ligand-receptor interaction	115	0.549806	both ends	1.19E-12

KEGG	ssc04640	Hematopoietic cell lineage	54	0.861014	bottom	5.83E-11
KEGG	ssc04514	Cell adhesion molecules	79	0.705132	bottom	1.67E-08
KEGG	ssc04061	Viral protein interaction with cytokine and cytokine receptor	48	0.928901	bottom	1.71E-08
KEGG	ssc04612	Antigen processing and presentation	39	1.07919	bottom	3.73E-08
KEGG	ssc05320	Autoimmune thyroid disease	23	1.41162	bottom	1.71E-07
KEGG	ssc03010	Ribosome	73	0.362753	bottom	2.3E-07
KEGG	ssc04940	Type I diabetes mellitus	26	1.05494	bottom	9.69E-07
KEGG	ssc05330	Allograft rejection	23	1.10792	bottom	2.04E-06
KEGG	ssc05323	Rheumatoid arthritis	55	0.786768	bottom	2.39E-06
KEGG	ssc05340	Primary immunodeficiency	21	1.0191	bottom	3.81E-06
KEGG	ssc05140	Leishmaniasis	43	0.791122	bottom	4.06E-06
KEGG	ssc05332	Graft-versus-host disease	22	1.08381	bottom	4.39E-06
KEGG	ssc05321	Inflammatory bowel disease	42	0.86574	bottom	4.75E-06
KEGG	ssc04672	Intestinal immune network for IgA production	31	1.00093	bottom	7.04E-06
KEGG	ssc05144	Malaria	32	0.651095	bottom	1.02E-05
KEGG	ssc04380	Osteoclast differentiation	70	0.521587	bottom	8.25E-05
KEGG	ssc05150	Staphylococcus aureus infection	40	0.618353	bottom	0.00025
KEGG	ssc04062	Chemokine signaling pathway	108	0.437426	bottom	0.00026
KEGG	ssc04659	Th17 cell differentiation	72	0.555607	bottom	0.00064
KEGG	ssc04064	NF-kappa B signaling pathway	69	0.446485	bottom	0.00093
KEGG	ssc04650	Natural killer cell mediated cytotoxicity	55	0.362997	bottom	0.0013
KEGG	ssc05416	Viral myocarditis	36	0.706473	bottom	0.0013
KEGG	ssc04610	Complement and coagulation cascades	46	0.556741	bottom	0.0036
KEGG	ssc05152	Tuberculosis	96	0.422057	bottom	0.0049
KEGG	ssc04142	Lysosome	83	0.374474	bottom	0.0052
KEGG	ssc05322	Systemic lupus erythematosus	33	0.695306	bottom	0.0054
KEGG	ssc04742	Taste transduction	28	0.556304	top	0.0056
KEGG	ssc04658	Th1 and Th2 cell differentiation	59	0.523849	bottom	0.0057
KEGG	ssc05168	Herpes simplex virus 1 infection	171	0.221474	bottom	0.006
KEGG	ssc00020	Citrate cycle (TCA cycle)	25	0.401737	top	0.0063
KEGG	ssc01200	Carbon metabolism	76	0.203544	top	0.0077
KEGG	ssc05145	Toxoplasmosis	70	0.451956	bottom	0.0083
Reactome	SSC-373076	Class A/1 (Rhodopsin-like receptors)	83	0.681523	bottom	2.26E-07

Reactome	SSC-72689	Formation of a pool of free 40S subunits	33	0.606559	bottom	2.26E-07
Reactome	SSC-72706	GTP hydrolysis and joining of the 60S ribosomal subunit	26	0.711684	bottom	9.94E-07
Reactome	SSC-500792	GPCR ligand binding	91	0.625133	bottom	1.64E-06
Reactome	SSC-156827	L13a-mediated translational silencing of Ceruloplasmin expression	40	0.526397	bottom	1.75E-06
Reactome	SSC-72737	Cap-dependent Translation Initiation	42	0.509061	bottom	1.94E-06
Reactome	SSC-1799339	SRP-dependent cotranslational protein targeting to membrane	29	0.60982	bottom	1.84E-05
Reactome	SSC-380108	Chemokine receptors bind chemokines	28	1.05443	bottom	1.84E-05
Reactome	SSC-68886	M Phase	169	0.188098	top	1.84E-05
Reactome	SSC-975956	Nonsense Mediated Decay (NMD) independent of the Exon Junction Complex (EJC)	31	0.55214	bottom	1.84E-05
Reactome	SSC-388396	GPCR downstream signalling	191	0.292799	both ends	7.47E-05
Reactome	SSC-375276	Peptide ligand-binding receptors	45	0.804189	bottom	8.65E-05
Reactome	SSC-975957	Nonsense Mediated Decay (NMD) enhanced by the Exon Junction Complex (EJC)	38	0.456397	bottom	0.00012
Reactome	SSC-68877	Mitotic Prometaphase	100	0.218568	top	0.00058
Reactome	SSC-72649	Translation initiation complex formation	29	0.406718	bottom	0.00067
Reactome	SSC-72702	Ribosomal scanning and start codon recognition	28	0.399524	bottom	0.0014
Reactome	SSC-2555396	Mitotic Metaphase and Anaphase	108	0.188481	top	0.0016
Reactome	SSC-72662	Activation of the mRNA upon binding of the cap-binding complex and eIFs, and subsequent binding to 43S	30	0.39362	bottom	0.0016
Reactome	SSC-68882	Mitotic Anaphase	107	0.186734	top	0.0019
Reactome	SSC-2467813	Separation of Sister Chromatids	84	0.183559	top	0.0078
COMPARTMENTS	GOCC:0022626	Cytosolic ribosome	66	0.693969	bottom	3.48E-21
COMPARTMENTS	GOCC:0022625	Cytosolic large ribosomal subunit	34	0.682204	bottom	5.67E-10
COMPARTMENTS	GOCC:0022627	Cytosolic small ribosomal subunit	31	0.762413	bottom	5.67E-10
COMPARTMENTS	GOCC:0044391	Ribosomal subunit	114	0.400617	bottom	2.54E-09
COMPARTMENTS	GOCC:0005840	Ribosome	120	0.356341	bottom	3.26E-08
COMPARTMENTS	GOCC:0009986	Cell surface	196	0.36959	bottom	3.26E-08
COMPARTMENTS	GOCC:0015935	Small ribosomal subunit	47	0.46814	bottom	5.87E-05

COMPARTMENTS	GOCC:1902495	Transmembrane transporter complex	125	0.367628	both ends	0.00035
COMPARTMENTS	GOCC:0015934	Large ribosomal subunit	69	0.348649	bottom	0.00046
COMPARTMENTS	GOCC:0034702	Ion channel complex	108	0.33378	top	0.00086
COMPARTMENTS	GOCC:0042788	Polysomal ribosome	22	0.38904	bottom	0.00089
COMPARTMENTS	GOCC:0034703	Cation channel complex	91	0.382755	top	0.0027
COMPARTMENTS	GOCC:0034705	Potassium channel complex	31	0.388139	both ends	0.0027
COMPARTMENTS	GOCC:0031012	Extracellular matrix	159	0.096372	bottom	0.0036
COMPARTMENTS	GOCC:0000793	Condensed chromosome	78	0.297318	top	0.0043
COMPARTMENTS	GOCC:0030141	Secretory granule	162	0.219564	both ends	0.0062
COMPARTMENTS	GOCC:0098552	Side of membrane	122	0.248387	bottom	0.0078
UniProt Keywords	KW-0407	Ion channel	82	0.583416	both ends	2.02E-09
UniProt Keywords	KW-0687	Ribonucleoprotein	108	0.287224	bottom	2.02E-09
UniProt Keywords	KW-0689	Ribosomal protein	77	0.3717	bottom	2.02E-09
UniProt Keywords	KW-0406	Ion transport	152	0.374323	both ends	7.58E-08
UniProt Keywords	KW-0297	G-protein coupled receptor	101	0.419511	both ends	7.65E-06
UniProt Keywords	KW-0807	Transducer	126	0.373869	both ends	1.28E-05
UniProt Keywords	KW-0851	Voltage-gated channel	28	0.591539	both ends	1.77E-05
UniProt Keywords	KW-0202	Cytokine	38	0.76108	bottom	3.37E-05
UniProt Keywords	KW-0505	Motor protein	62	0.396312	top	0.000034
UniProt Keywords	KW-0391	Immunity	57	0.614779	bottom	0.0012
UniProt Keywords	KW-0436	Ligase	50	0.299495	top	0.0012
UniProt Keywords	KW-0633	Potassium transport	29	0.488758	both ends	0.0012
UniProt Keywords	KW-0393	Immunoglobulin domain	78	0.371926	bottom	0.0018
UniProt Keywords	KW-0853	WD repeat	174	0.205994	top	0.0034
UniProt Keywords	KW-0130	Cell adhesion	59	0.449967	bottom	0.0043
UniProt Keywords	KW-0630	Potassium	36	0.377132	both ends	0.0052
UniProt Keywords	KW-0165	Cleavage on pair of basic residues	31	0.537739	both ends	0.0057
Pfam	PF10320	Serpentine type 7TM GPCR chemoreceptor Srsx	50	0.701476	both ends	1.1E-07
Pfam	PF00001	7 transmembrane receptor (rhodopsin family)	114	0.508672	bottom	1.36E-07
Pfam	PF07654	Immunoglobulin C1-set domain	33	1.1711	bottom	7.91E-07
Pfam	PF07686	Immunoglobulin V-set domain	113	0.544085	bottom	1.58E-06

Pfam	PF13927	Immunoglobulin domain	166	0.318591	bottom	2.66E-05
Pfam	PF00047	Immunoglobulin domain	153	0.316201	bottom	8.46E-05
Pfam	PF00520	Ion transport protein	49	0.565514	both ends	0.00013
Pfam	PF13895	Immunoglobulin domain	125	0.365916	bottom	0.00048
Pfam	PF00059	Lectin C-type domain	38	0.730318	both ends	0.0024
Pfam	PF00271	Helicase conserved C-terminal domain	83	0.268068	top	0.0042
InterPro	IPR017452	GPCR, rhodopsin-like, 7TM	111	0.480819	both ends	2.02E-08
InterPro	IPR000276	G protein-coupled receptor, rhodopsin-like	110	0.484113	both ends	2.46E-08
InterPro	IPR003597	Immunoglobulin C1-set	26	1.37608	bottom	5.14E-06
InterPro	IPR013106	Immunoglobulin V-set domain	97	0.602733	bottom	1.05E-05
InterPro	IPR005821	Ion transport domain	50	0.525875	both ends	0.000017
InterPro	IPR003006	Immunoglobulin/major histocompatibility complex, conserved site	23	1.25762	bottom	7.16E-05
InterPro	IPR011989	Armadillo-like helical	152	0.247023	top	7.16E-05
InterPro	IPR036961	Kinesin motor domain superfamily	53	0.459247	top	0.00031
InterPro	IPR011162	MHC classes I/II-like antigen recognition protein	22	1.12071	bottom	0.0017
InterPro	IPR008983	Tumour necrosis factor-like domain superfamily	29	0.792984	both ends	0.0038
InterPro	IPR027359	Voltage-dependent channel domain superfamily	23	0.5187	both ends	0.0038
InterPro	IPR001304	C-type lectin-like	39	0.318704	bottom	0.0074
InterPro	IPR014001	Helicase superfamily 1/2, ATP-binding domain	83	0.272641	top	0.0074
InterPro	IPR016186	C-type lectin-like/link domain superfamily	47	0.193553	bottom	0.0074
InterPro	IPR001650	Helicase, C-terminal	82	0.247572	top	0.0094
SMART	SM00409	Immunoglobulin	190	0.55623	bottom	1.14E-13
SMART	SM00407	Immunoglobulin C-Type	28	1.28552	bottom	4.38E-07
SMART	SM00034	C-type lectin (CTL) or carbohydrate-recognition domain (CRD)	39	0.721911	both ends	0.00086
SMART	SM00487	DEAD-like helicases superfamily	77	0.270194	top	0.0049
SMART	SM00490	Helicase superfamily c-terminal domain	77	0.269953	top	0.0058

Suppl. Table 5: Missense variants in RFI QTL genes.

Uploaded_variation	Location	SYMBOL	Amino acids	SIFT	Conserved domain
9_86549936_A/C	9:86549936	*AHR*	T/P	1	-
9_86550830_G/T	9:86550830	*AHR*	G/C	0,3	-
9_86551088_T/C	9:86551088	*AHR*	F/L	1	-
9_86553513_A/C	9:86553513	*AHR*	T/P	1	-
9_88762764_G/T	9:88762764	*POLR1F*	T/K	1	-
9_89160365_C/T	9:89160365	*MACC1*	V/I	0,28	-
9_90767111_G/C	9:90767111	*DNAH11*	R/T	0	Dynein_heavy
9_90767143_G/A	9:90767143	*DNAH11*	A/T	0	Dynein_heavy
9_90774352_C/T	9:90774352	*CDCA7L*	R/K	0,79	-
9_90778151_A/G	9:90778151	*CDCA7L*	S/P	1	-
9_90962105_T/C	9:90962105	*RAPGEF5*	I/V	1	RasGEF
